# TM4SF1 upregulates MYH9 to activate the NOTCH pathway to promote cancer stemness and lenvatinib resistance in HCC

**DOI:** 10.1186/s13062-023-00376-8

**Published:** 2023-04-17

**Authors:** Si-bo Yang, Zi-han Zhou, Jin Lei, Xiao-wen Li, Qian Chen, Bo Li, Ye-wei Zhang, Yu-zhen Ge, Shi Zuo

**Affiliations:** 1 Department of Clinical Medicine, Guizhou Medical University, No. 9 Beijing Road, Yunyan District, Guiyang, 550001 Guizhou People’s Republic of China; 2 Department of Hepatobiliary Surgery, The Affiliated Hospital of Guizhou Medical University, Guiyang, 550001 Guizhou People’s Republic of China; 3 Dalian University Medical College, No. 10 Xuefu Street, Dalian, 116622 Liaoning People’s Republic of China; 4 Department of Organ Transplant, The Affiliated Hospital of Guizhou Medical University, Guiyang, 550001 Guizhou People’s Republic of China

## Abstract

TM4SF1, a member of the transmembrane 4 superfamily, is crucial for both healthy and malignant human tissues. The significant function of TM4SF1 in the incidence and progression of cancer has been widely recognized in recent years. Although some achievements have been made in the study of TM4SF1, the effect of TM4SF1 on cancer stemness in hepatocellular carcinoma (HCC) and its molecular basis are yet to be reported. We found through abundant in vitro and in vivo experiments which the expression of TM4SF1 was positively correlated with the progression and cancer stemness of HCC. We identified the downstream protein MYH9 of TM4SF1 and its final regulatory target NOTCH pathway using bioinformatics analysis and protein mass spectrometry. We cultivated a Lenvatinib-resistant strain from HCC cells to examine the relationship between cancer stemness and tumor drug resistance. The study confirmed that TM4SF1 could regulate the NOTCH pathway by upregulating MYH9, thus promoting cancer stemness and Lenvatinib resistance in HCC. This study not only provided a new idea for the pathogenesis of HCC but also confirmed that TM4SF1 might become a new intervention point to improve the clinical efficacy of Lenvatinib in treating HCC.

**Supplementary Information:**

The online version contains supplementary material available at 10.1186/s13062-023-00376-8.

## Introduction

With 905,677 individuals, liver cancer rose to the sixth-most prevalent cancer worldwide in 2020. The number of people dying from liver cancer also reached 830,180, ranking third among all cancers [1]. The majority (80%) of initial liver cancers are hepatocellular carcinomas (HCC) [2]. The mechanism of HCC's occurrence and progression still needs to be investigated, despite recent advancement in research and treatment. Previous studies showed that the occurrence and development of HCC in many cases were caused by the imbalance of signal pathways instigated by gene mutations [3], including upregulations of Wnt/β-catenin, Akt/mTOR, P53 signal pathway [4] and so on. An in-depth exploration of these processes can help us to treat HCC more specifically. Moreover, some scholars have found that cancer stem cells (CSCs) are a major factor affecting tumor recurrence and metastasis [5, 6], which has also gained our attention. CSCs are a kind of cells with multi-directional differentiation potential and unlimited proliferation. Their division and differentiation are not regulated by the body [7]. They are also considered one of the principal sources of cancer [8], as tumors continue to grow due to the continuous self-renewal and differentiation of CSCs. Therefore, the study of cancer stemness has clinical value and profound clinical significance [9]. The conclusion that CSCs can effectively promote drug resistance in tumors has been widely accepted [10–12]. Among the first-line drugs used for treating HCC [13, 14], Lenvatinib is the first targeted drug approved for advanced-stage HCC and plays a considerable role in clinical treatment. However, it still cannot prevent patients from developing drug resistance, resulting in a poor prognosis [15]. At present, different opinions are available on the molecular mechanism of these phenomena, leading to increasing attention from scientists for further research. TM4SF1 is located in 3q25.1 and has four highly conserved transmembrane domains, two extracellular rings, and one intracellular small ring [16, 17]. Tumor-associated antigen was how TM4SF1 was initially classified. It stabilizes the cell signal complex and plays a role in cell proliferation, adhesion, and movement [18, 19]. Studies have confirmed that TM4SF1 is upregulated in a variety of cancers [20–23] including HCC. A study by Tang and colleagues in 2020 showed that TM4SF1 might also have a potential effect on cancer stemness [24]. Using the gene set enrichment analysis (GSEA) technology, we enriched the signaling pathways significantly related to TM4SF1 and found that the NOTCH pathway was related to cancer stemness. After protein mass spectrometry analysis of TM4SF1, we screened out the downstream protein MYH9, which, as a widely expressed cytoskeletal protein, was closely related to a variety of cancers. Hence, we can assume that TM4SF1 may activate the NOTCH pathway via upregulation of MYH9, ultimately promoting tumor stemness in HCC. Moreover, TM4SF1 may also affect the resistance of HCC to Lenvatinib and become a potential target for the clinical treatment of HCC.

## Materials and methods

### Cell culture

Five HCC cell lines and human normal liver cell LO2 were employed in this study. All HCC cell lines (Hep-G2, Hep-3B, LM3, Huh7, and MHCC97H) were acquired from the Chinese Academy of Sciences' cell bank, while LO2 was acquired from Southern Medical University's Cancer Research Institute. All cells were grown in Dulbecco's modified Eagle's medium (DMEM) with 10% fetal bovine serum (FBS) from GIBCO, NY, USA, and antibiotics from BIOMYC-3 Antibiotic Solution and Penicillin–Streptomycin Amphotericin B Solution from Biological Industries, Israel, at 37 °C and 5% CO_2_.

### Clinical specimens and immunohistochemistry (IHC)

We purchased a HCC tissue microarrays from Shanghai Outdo Biotech Company, containing 90 samples of HCC and 90 samples of paracancerous tissues. The clinical samples were approved by patients' informed consent and authoritative histopathologists, and passed the review of the ethics committee on the use of the samples (Ctl No.:YB M-05-02). After a series of steps such as dewaxing, hydration, block the activity of endogenous peroxidase and nonspecific antigen, antigen retrieval, incubation with first antibody, staining and sealing. The tissue microarray was observed under Inverted microscope and double-blind scored in accordance with staining intensity (0-Colorless, 1-light yellow, 2-light brown, 3-dark brown) and positive area ratio (0: < 5%, 1:5–25%, 2:25–50%, 3:50–75%, 4: > 75%) by The Department of Pathology of the Hospital of Integrated traditional Chinese and Western Medicine of Southern Medical University (Score calculation: staining intensity grade*positive area proportion grade).

### SiRNA, plasmids and transfection conditions

The cells to be transfected were cultured in cell petri dishes for 48–72 h in advance. SiRNA (Ribo-Bio, Guangzhou, China) and overexpressing plasmid (GeneChem, Shanghai, China) was introduced into cells by Lipofectamine 3000 (Thermo Fisher Scientific Company, USA) to obtain cell lines that temporarily silenced TM4SF1 or MYH9 (Additional file 1: Table S1).

### Lentivirus production and infection

Lentivirus human TM4SF1 gene was introduced into lentiviral vector GV367 to construct lentiviral TM4SF1 (LV-TM4SF1) (GeneChem, Shanghai, China) to infect hepatoma cells, and stable hepatoma cell lines with loss of TM4SF1 expression was constructed. On this basis, the empty vector lentivirus (LVNC) (GeneChem, Shanghai, China) was used as the control, and the cells were screened with puromycin, and the polyclonal cells with green fluorescence signal were selected for follow-up experiment (Additional file 1: Table S1).

### Extraction of RNA and QPCR

The total RNA of cells was obtained by using RNA extraction kit (Foregene, Chengdu, China), and cDNA was amplified by reverse transcription kit produced by Takara company. Finally, quantitative polymerase chain reaction of cDNA was performed with the help of Bio-Rad T100 system using primers ordered from Guangzhou IGE Biology Company (Additional file 1: Table S2), and the changes of genes were detected by calculating the data.

### Western blot analysis and antibody

The protein of the cell was obtained from the solution system of lytic buffer, protease inhibitor in the ratio of 100:1:1 and phosphatase inhibitor. The pure protein was collected by ultrasound and centrifugation and quantified by BCA protein analysis kit. SDS–polyacrylamide gel electrophoresis was used to separate the protein. It was then moved to a polyvinylidene fluoride membrane and treated at 4 °C with the desired primary antibody. The antibodies used in the experiment include TM4SF1, CD44, CD133, OCT4, SOX2, MYH9, NOTCH1, JAGGED1, HES1, β-Tubulin (Additional file 1: Table S3). After the protein was fully combined with the primary antibody, the chemiluminescence kit (Thermo Fisher Scientific Company, USA) was used to image in the ChemiDoc XRS + molecular imager (Bio-Rad, Hercules, CA, USA)to analyze the expression of the protein.

### Tumour sphere formation

The cells (5 × 10^3^) were inoculated into six-well ultra-low attachment plates per well (Corning, NY, USA) and cultured with special sphere formation medium (It is composed of 1 mL B27, 20 uL human EGF of 20 ng/mL and 20 uL human FGF of 20 ng/mL added to 50 mL DMEM/F12 culture medium) at 37 °C, 5% CO_2_ for 1–2 weeks. Tumour spheres were observed and photographed with inverted microscope. The proportions of tumour spheres with diameters of 50–100 μm, 100–150 μm and > 150 μm in the total visual field were calculated, and statistical charts were drawn.

### Detection of side population cells

After the experimental cells were taken out for digestion and centrifugation, the supernatant was abandoned and the cells were re-suspended with 1 mL high-glucose medium containing 2% serum. After cell counting, the cell concentration was adjusted to 1 × 10^6^ cells/mL in high-glucose medium containing 2% serum heated by 37 °C water bath, and then transferred into the flow tube. A blank control group was set for each experimental group, plus 50 μM verapamil. After covering the lid, it was first incubated in a 37 °C water bath for 30 min. The flow tube was taken out every 10 min, mixed up and down gently once, centrifuged at 12000 r/min and 4 °C for 5 min, then the supernatant was discarded, and the medium was added for re-suspension. One of the samples was added with DCV dye solution and the concentration was adjusted to 20 uM. After covering the sample, it was placed in a 37 °C water bath and incubated for 90 min to make it fully stained. The flow tube was taken out every 10 min, gently mixed up and down once, centrifuged at 12000 r/min and 4 °C for 5 min, and the supernatant was discarded. The PBS cells were precooled at 4 °C and detected by upflow cytometry after treatment.

### Fluorescence-activated cell sorting

Consistent with the above procedure for “3.8 Detection of Side population cells”, after the cells were suspended by PBS, 5μLPI was added into the cell suspension under the condition of avoiding light. Note that the experimental process was carried out on the ice box. After the operation, upflow cytometry was used to detect the expression of side population cells, and then FACS staining was performed. Co-incubated with FITCCD133, PE-CD44 antibody, and AF647 sheep anti-mouse immunoglobulin antibody (AF647), staining cells were taken for LSRFortessa or AriaII (BD) analysis or classification.

### Protein mass spectrometry

The protein extracted from the tested cells was entrusted to Fitgene Biotechnology company (Guangzhou, China)for protein spectrum analysis, and the genes whose expression degree changed after silencing TM4SF1 were screened. These genes were further screened according to the multiple of gene expression difference and bioinformatics database search, the appropriate genes were determined for further research.

### Coimmunoprecipitation (co-IP)

The experimental cells were removed and lysed with mild lysate. The cell suspension was transferred to a 1.5mLEP tube, centrifuged at 4 °C and 12000r for 30 min, supernatant was taken, and a small amount of protein samples were taken to a new 1.5mLEP tube and stored at − 20 °C for protein western blotting analysis. The remaining protein samples were added to the protein primary antibody of the decoy protein and incubated overnight with slow shaking at 4 °C. Pretreated 10 μL proteinA agarose beads were added to the protein samples incubated with the antibody overnight. The samples were slowly shaken at 4 °C for 2–4 h to conjugate the antibody to the proteinA agarose beads. After the immunoprecipitation reaction, the agar–agar beads were centrifuged at 4 °C and 3000r for 3 min to the bottom of the tube, the supernatant was absorbed, and the agar–agar beads were washed with 1 mL cracking buffer for 3–4 times. Adding 15μL loadingbuffer sample buffer, boiling water for 5 min, finally by western blot analysis.

### Establishment of Lenvatinib-resistant cell lines

The cells were cultured for a long time by the method of increasing concentration, and the model of Lenvatinib-resistant strain was established. At 37 °C and 5% CO_2_, the cells were grown in DMEM containing 10% fetal bovine serum (FBS). Lenvatinib of 1 mg was administered to the culture media once the cells had stabilized. After 1 week of culture, if the cells could grow stably, they would be changed to a higher concentration of Lenvatinib (increasing 1 mg each time), until the cells could grow stably in the culture medium containing 10 mg Lenvatinib, indicating that Lenvatinib-resistant cells were induced successfully.

### Etermination of CCK-8 cell viability

The cell survival rate and tolerance to Lenvatinib were detected by CCK-8 method. According to previous studies [25], a 96-well plate was prepared, the experimental cells were taken out for digestion, centrifugation and suspension, 5000 cells were counted by cell counting method, diluted with 100 μL medium and inoculated into each pore, then placed back into the incubator for 24 h. After observing the cell growth status, 10 μL of different concentrations of Lenvatinib were added into each pore and placed back into the incubator for 48 h. The 96-well plates were taken out and added with 10 μL CCK-8 reagent in each well under the condition of hiding from light. The 96-well plates were wrapped with aluminum foil and put back into the incubator for further culture for 1–4 h. The 96-hole plate was removed, the lid was opened, and OD values were measured at 450 nm wavelength with enzyme marker. Finally, standard curves were drawn with the help of Graphpadprism software.

### Mouse xenografts

All animal experiments meet the requirements of the International guidelines for Animal Care and Conservation and the Animal Research Committee of the academic Medical Center of Southern Medical University. The tumor growth was evaluated by subcutaneous transplantation of xenogeneic oysters in mice.

LM3 with stable expression loss of TM4SF1(LV-TM4SF1) in different concentration gradients was subcutaneously injected into 3 week-old male nude mice weighing 16–25 g, and the corresponding concentration of LM3 infected with the empty vector lentivirus (LV-NC) was used as negative control. The growth of the tumor was observed and the size of the tumor was recorded with Vernier caliper every three days. 45 days later, the tumor was taken out, photographed and weighed, and the data of the tumor were collected for statistics and analysis.

The effect of TM4SF1 on drug resistance of human hepatocellular carcinoma cell line Lenvatinib was studied by subcutaneous transplantation in nude mice. Saline containing Lenvatinib was injected into nude mice by intraperitoneal injection in advance. The experimental group of nude mice received subcutaneous injections of various quantities of Lenvatinib-resistant LM3; untreated LM3 served as the negative control. Then, according to the previous method, nude mice were cultured and tumor data were collected for follow-up experiments.

### Primary cell culture

Fresh tumor tissue was taken, cut into small pieces with a diameter of about 4 mm, and repeatedly rinsed with PBS until the blood clots and adipocytes on the tissue fragments were basically removed, then transferred to a centrifuge tube, digested in a 37 °C water bath with 0.25% trypsin for 15–20 min, rinsed with PBS for 2–3 times, and then gently blown off the digested cells with a small amount of cell medium. Cells are counted and cultured at appropriate concentrations. Note that a sterile environment should be carefully maintained during the experiment to avoid cell contamination. If there is still undigested tissue, the above steps can be repeated for multiple digestion.

### Bioinformatics analysis

The study used bioinformatics databases to obtain materials that can support the conclusions of the study. The databases used include: The Cancer Genome Atlas (TCGA), TIMER2.0, Gene Expression Profiling Interactive Analysis (GEPIA). Gene Set Enrichment Analysis (GSEA) technique was used to enrich the KEGG and GO pathways of TM4SF1, and the pathways related to its expression were obtained.

### Statistical analysis

Each group of in vitro experiments were repeated three times to ensure the credibility and reproducibility of the study, and the average-standard deviation (x ± s) was used to express the measured results. SPSS13.0 software was used to analyze the statistical process and data in the study, and t-test was used to compare between groups, the difference was statistically significant (*P* < 0.05).

## Results

### High expression of TM4SF1 was closely related to the progression and poor prognosis of HCC

**Fig. 1:**
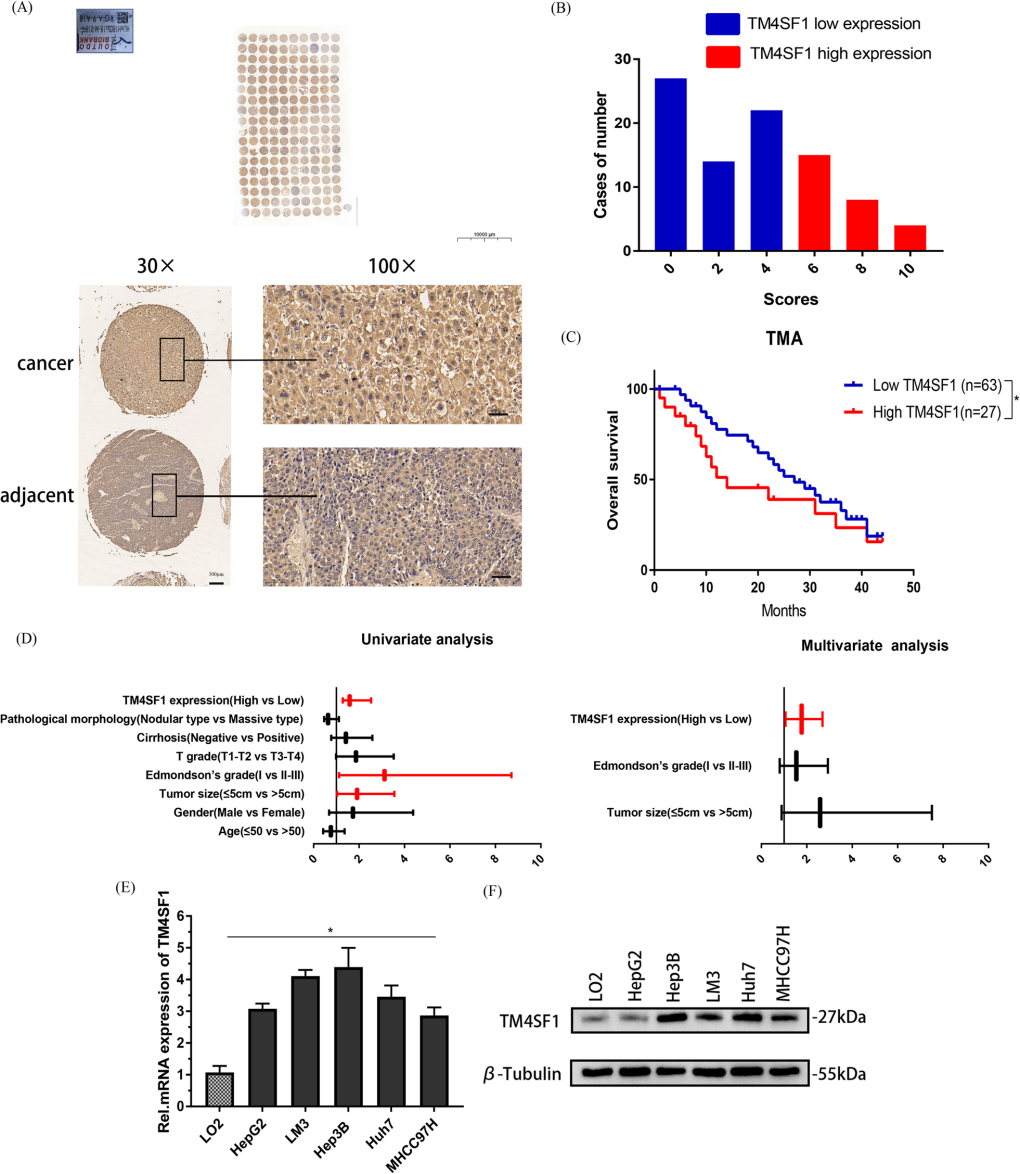
High levels of TM4SF1 expression are associated with poor prognosis in hepatocellular cancer. **A** Results of immunohistochemical analysis of HCC tissue microarray showed that the expression of TM4SF1 in HCC tissues increased significantly (*n* = 90). **B** Hepatocellular carcinoma tissues were graded according to IHC staining index. **C** Analysis of the 90 HCC patients revealed that patients with low TM4SF1 expression had a higher overall survival rate than patients with high TM4SF1 expression. **D** Univariate and multivariate Cox regression analysis of overall survival in 90 HCC patients. **E** According to the results of qPCR, HCC cells have significant levels of TM4SF1 expression. **F** Results of the WB analysis showed that TM4SF1 was highly expressed in HCC cells. ^*^P < 0.05; ^**^P < 0.01; ^***^P < 0.001

We found via the bioinformatics analysis using the TIMER2.0 and GEPIA databases in the early stage of the experiment that TM4SF1 was much more expressed in human HCC tissues than in healthy liver tissues (Additional file 2: Fig. S1A and B). TM4SF1 and the cumulative survival rate, overall survival rate, and disease-free survival rate in patients with HCC were further examined. Patients with strong TM4SF1 expression had a generally unfavorable prognosis, we found. (Additional file 2: Fig. S1C and D). We also measured the expression of TM4SF1 in all liver cancer cell lines using the TCGA database, and the results revealed that the majority of these cell lines had high levels of TM4SF1 expression (Additional file 2: Fig. S1E). Then, we purchased the tissue microarray with 90 hepatocellular carcinoma and paracancerous tissues for immunohistochemical experiment (Fig. 1A) and scored (Fig. 1B). The findings demonstrated that HCC tissues had much greater levels of TM4SF1 expression than paracancerous tissues. Additionally, utilizing the prognostic data from 90 patients with hepatocellular carcinoma, we examined the overall survival rate. The findings demonstrated that patients with low expression of TM4SF1 had a higher overall survival rate than those with high expression of TM4SF1 (Fig. 1C). And than, using SPSS 13.0 statistical software to analyze our data, we found that the expression of TM4SF1 was positively correlated with the tumor size, Edmondson–Steiner grade, and cirrhosis, but not with sex, age of the patients and so on (Table 1). The expression of TM4SF1, tumor size, and Edmondson-Steiner grade were found to be linked with the overall survival percentage of 90 HCC patients in both univariate and multivariate analyses (Table 2, Fig. 1D). We cultured five hepatoma cell lines (Hep3B, HepG2, LM3, Huh7, and MHCC97H) and normal liver cells LO2, extracted RNA and protein from the cells, and detected the expression of TM4SF1 at the RNA and protein levels using qPCR and Western blot (WB), respectively (Fig. 1E and F).

**Table 1:** The basic information of 90 patients with HCC

Clinicopathological features	Number	Low expression, N (%)	High expression, N (%)	*P* value
*Age*				
≤ 50	31	23	8	0.5292
> 50	59	40	19	
*Gender*				
Male	74	53	21	0.5503
Female	16	10	6	
*Tumor size*				
≤ 5 cm	44	37	7	0.0043**
> 5 cm	46	26	20	
*Edmondson’s grade*				
I	21	20	1	0.0039**
II–III	69	43	26	
*T grade*				
T1–T2	52	36	16	0.8516
T3–T4	38	27	11	
*Cirrhosis*				
Negative	73	55	18	0.0219*
Positive	17	8	19	
*Pathological morphology*				
Nodular type	54	37	17	0.7073
Massive type	36	26	10	

*P < 0.05 **P < 0.005

**Table 2:** Univariate and multivariate Cox regression analysis of overall survival in 90 HCC patients

Variables	Overall survival		
	HR	95 Cl	*P* value
*Univariate analysis*			
Age	0.759	0.422–1.366	0.358
Gender	1.730	0.683–4.383	0.248
Tumor size	1.913	1.029–3.555	0.040*
Edmondson’s grade	3.120	1.117–8.709	0.030*
T grade	1.864	0.986–3.523	0.055
Cirrhosis	1.417	0.775–2.588	0.257
Pathological morphology	0.637	0.462–1.116	0.692
TM4SF1 expression	1.587	1.283–2.531	0.019*
*Multivariate analysis*			
Tumor size	2.588	0.892–7.506	0.080
Edmondson’s grade	1.542	0.811–2.932	0.187
TM4SF1 expression	1.774	1.063–2.689	0.036*

*P < 0.05

### TM4SF1 upregulated the stemness of tumor cells in HCC

**Fig. 2:**
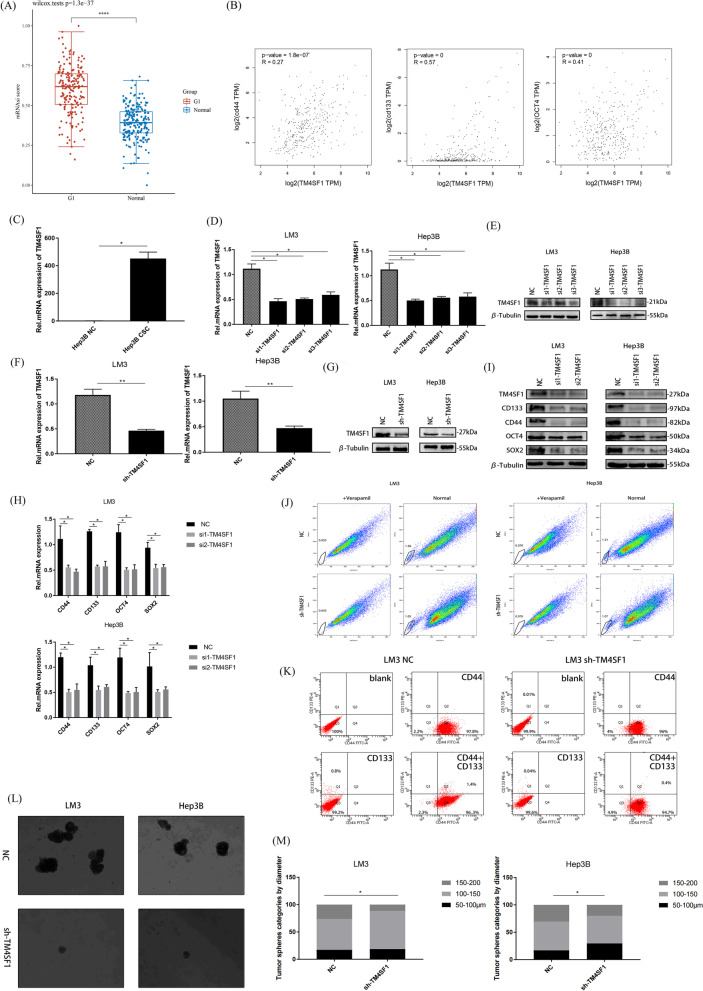
Silencing TM4SF1 could inhibit the cancer stemness in HCC in vitro. **A** Stemness index of TM4SF1 in 371 HCC samples from the TCGA database was calculated using the OCLR algorithm, and the degree of stemness correlation of TM4SF1 was evaluated. **B** The connection between TM4SF1 and chemicals associated to cancer stem cells was examined using the GEPIA database. **C** RNA was extracted from Hep3B tumor spheres and detected via qPCR. The expression of TM4SF1 in tumor balls abnormally increased. **D** qPCR was used to verify the knockout efficiency of TM4SF1 in Hep3B and LM3 after transient transfection. **E** WB was used to confirm the knockout efficiency of TM4SF1 in Hep3B and LM3 after transient transfection. **F** qPCR was used to verify the knockout efficiency of TM4SF1 in Hep3B and LM3 after lentivirus infection. **G** WB was used to verify the knockout efficiency of TM4SF1 in Hep3B and LM3 after lentivirus infection. **H** The outcomes of qPCR analysis revealed that TM4SF1 silencing reduced the expression of markers linked to cancer stem cells. **I** The outcomes of WB analysis revealed that TM4SF1 silencing reduced the expression of markers linked to cancer stem cells. **J** Results of the experiment for the detection of side-population cells showed that after silencing TM4SF1, the proportion of stem cells in hepatocellular carcinoma cells decreased significantly. **K** The ratio of CD44 positive and CC133 positive cells in TM4SF1 silenced LM3 and control group was determined by fluorescence-activated cell sorting. **L** Experiment for tumor sphere formation showed that the number and diameter of tumor spheres decreased after TM4SF1 was silenced. Scale bars, 50 μm. **M** Comparison of the number of tumor spheres and the proportion of tumor spheres with different diameters. ^*^P < 0.05; ^**^P < 0.01; ^***^P < 0.001

In order to prove the correlation between TM4SF1 and cancer stemness, we used OCLR algorithm to evaluate the stemness index of TM4SF1 based on the tissues of 371 patients with HCC in TCGA database. In HCC tissues compared to normal tissues, it was discovered that TM4SF1 had a greater stemness index (Fig. 2A). Using the GEPIA database, we examined the relationship between TM4SF1 and molecules associated with cancer stem cells. We discovered that TM4SF1 expression was positively correlated with CD44, CD133, and OCT4 (Fig. 2B). We verified the high expression of TM4SF1 in tumor spheres via the PCR experiment using the RNA extracted from the tumor spheres of liver cancer provided by the Cancer Research Institute of Southern Medical University, Guangzhou, China (Fig. 2C). Then, we selected two HCC cell lines Hep3B and LM3 as the research objects and inserted siRNA into the cells to silence TM4SF1 with the help of a transfection reagent Lipofectamine 3000, and verify the efficiency of silencing. (Fig. 2D and E). The relationship between TM4SF1 and cancer stem–related molecules CD44, CD133, OCT4, and SOX2 was detected via qPCR and WB, which confirmed the decrease in the expression of CD44, CD133, OCT4, and SOX2 with the silencing of TM4SF1 (Fig. 2F and G). To investigate the impact of TM4SF1 on the stem cell-related phenotype of HCC, we infected Hep3B and LM3 cells with lentiviruses carrying silenced portions of TM4SF1. After verifying the efficiency (Fig. 2D and E), The side-population cell detection test revealed that the proportion of CSCs was also lower in HCC cells silenced by TM4SF1 than in the control group (Fig. 2H). We confirmed via tumor sphere formation and side-population cell detection tests that TM4SF1 could affect the cancer stemness in HCC. The results showed that the size and number of tumor spheres formed by TM4SF1-silenced HCC cells were inferior to those in the control group (Fig. 2I and J). In order to further confirm whether TM4SF1 is related to the cancer stem of HCC, we isolated CD133+, CD44+ and CD133+/CD44+ cells from LM3 cells with silenced TM4SF1 and LM3 cells from untreated group by flow cytometry. It showed that the proportion of CD133+, CD44+ and CD133+/CD44+ cells in the silent TM4SF1 group was inferior to which in the control group (Fig. 2K).

**Fig. 3:**
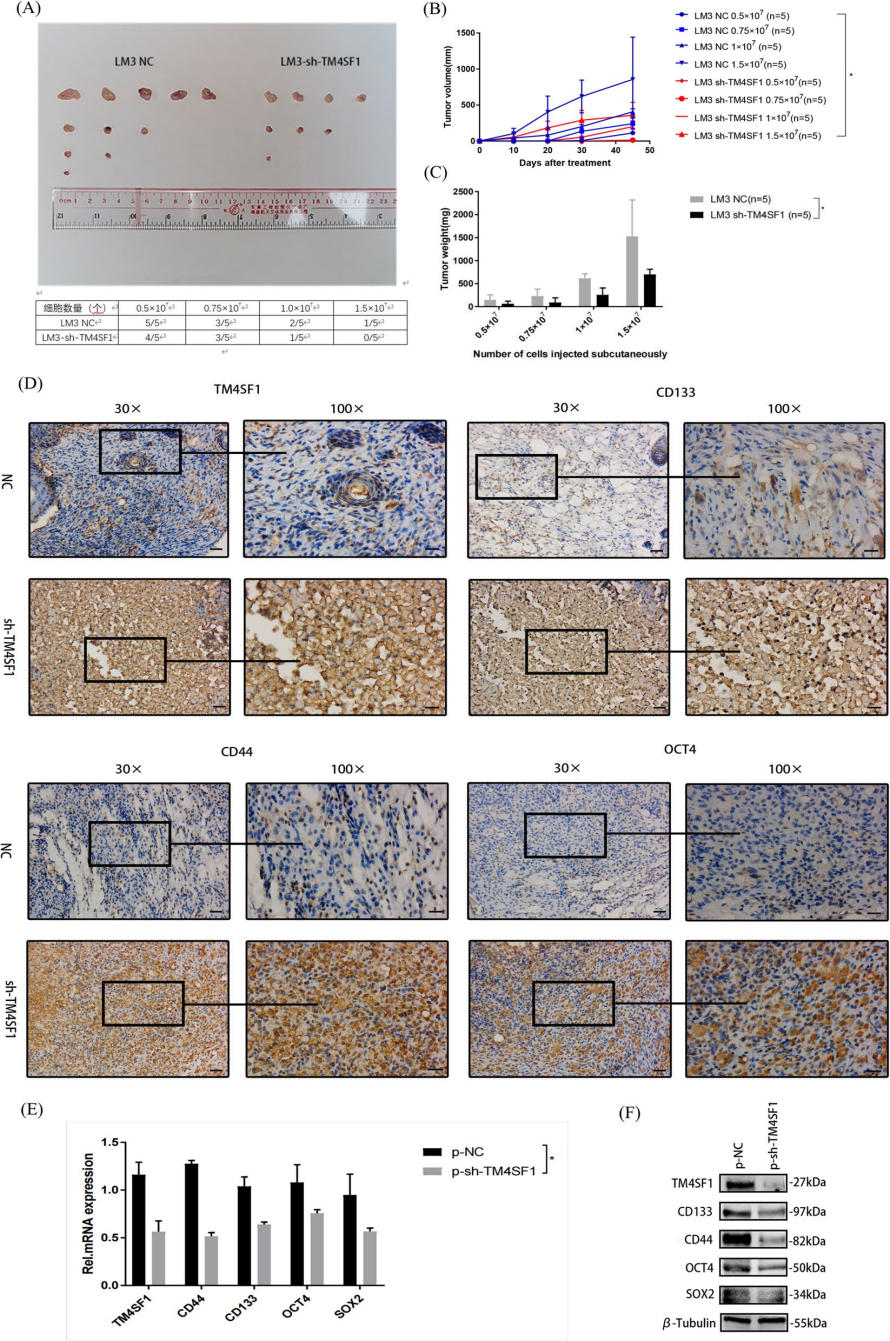
Silencing TM4SF1 could inhibit the growth and stemness in HCC in vivo. **A** Subcutaneous tumor removed from nude mice after 45 days of culture. **B** Statistical comparison of tumor volume between knockdown TM4SF1 and control groups. **C** Statistical comparison of tumor weight between knockdown TM4SF1 and control groups. **D** Immunohistochemical results of subcutaneous tumors in nude mice showed that after silencing TM4SF1, the expression of cancer stem–related molecules decreased with the decrease in TM4SF1 expression. Scale bars, 30 ×: 300 μm, 100 ×: 100 μm. **E** RNA was extracted from primary cultured tumor cells of nude mice. The qPCR analysis showed that the expression of cancer stem–related molecules decreased after silencing TM4SF1. **F** Results of the WB analysis showed that after TM4SF1 silencing, the expression of cancer stem–related molecule decreased in primary tumor cells of nude mice. ^*^P < 0.05; ^**^P < 0.01; ^***^P < 0.001

When carrying out the in vitro experiment, we also considered the subcutaneous tumorigenesis experiment of nude mice as the in vivo experiment to verify the influence of TM4SF1 on the occurrence and progression of HCC. The nude mice in the experimental group were administered by injection with different numbers of LM3 cells infected with LV-TM4SF1; the LM3 cells infected with no-load Lentivirus were used as the control. The changes in size during tumor growth were recorded, and the tumor was removed 45 days later (Fig. 3A). However, we found that the tumor size and weight in the experimental group were smaller than those in the control group regardless of the number of days or the number of cells (Fig. 3B and C). We also made tissue sections of subcutaneous tumors of nude mice in previous experiments and performed immunohistochemical staining. We chose TM4SF1, CD44, CD133, and OCT4 as the primary antibodies to combine with tumor tissues. It demonstrated that, compared to the control group, the staining score in the experimental group was much lower (Fig. 3D). We grew primary cells from the collected tumor tissues of nude mice. After the cells grew stably, RNA and protein were extracted and qPCR and WB were performed. The results showed that the expression of TM4SF1 and stem-related molecules in primary tumor cells of nude mice increased (Fig. 3E and F).

### TM4SF1 activated the NOTCH pathway and promoted cancer stemness in HCC cells by upregulating MYH9

**Fig. 4:**
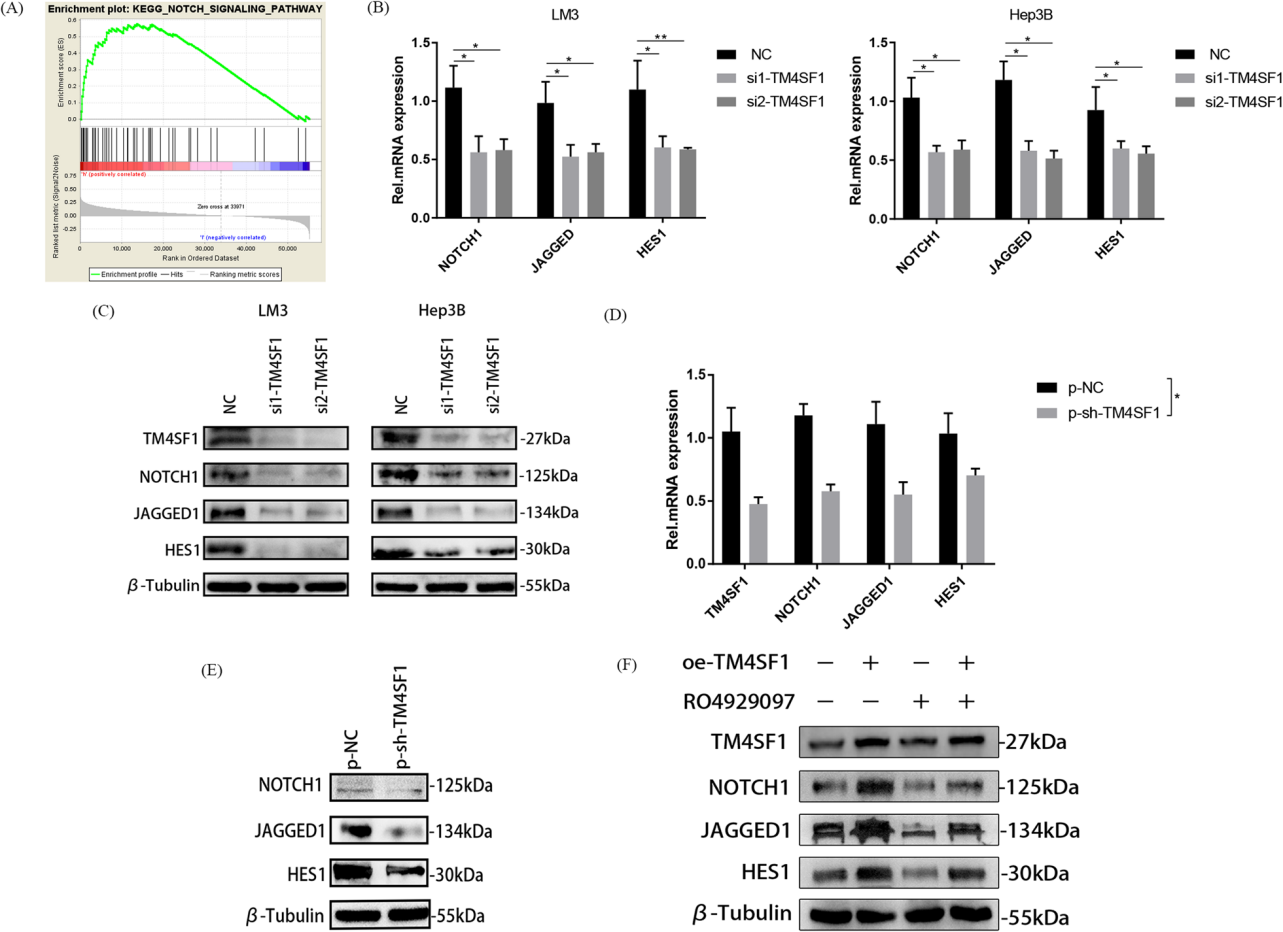
TM4SF1 could activate the NOTCH pathway to promote cancer stemness. **A** The cancer stem–related NOTCH pathway was obtained via the enrichment of the TM4SF1-related pathways using the GSEA technology. **B** The qPCR analysis revealed that after TM4SF1 was silenced, the expression of molecules connected to the NOTCH pathway reduced. **C** The WB analysis revealed that after TM4SF1 was silenced, the expression of molecules connected to the NOTCH pathway reduced. **D** Primary grown tumor cells from nude mice were used to harvest RNA. Following the silencing of TM4SF1, the qPCR analysis revealed the expression of molecules connected to the NOTCH pathway. **E** The results of the WB analysis demonstrated that, following TM4SF1 silencing, primary tumor cells from nude mice expressed chemicals relevant to the NOTCH pathway. **F** WB analysis showed that overexpression of TM4SF1 could reverse the inhibitory effect of the NOTCH pathway targeted inhibitor RO4929097 on the molecules connected to the NOTCH pathway. ^*^P < 0.05; ^**^P < 0.01; ^***^P < 0.001

**Fig. 5:**
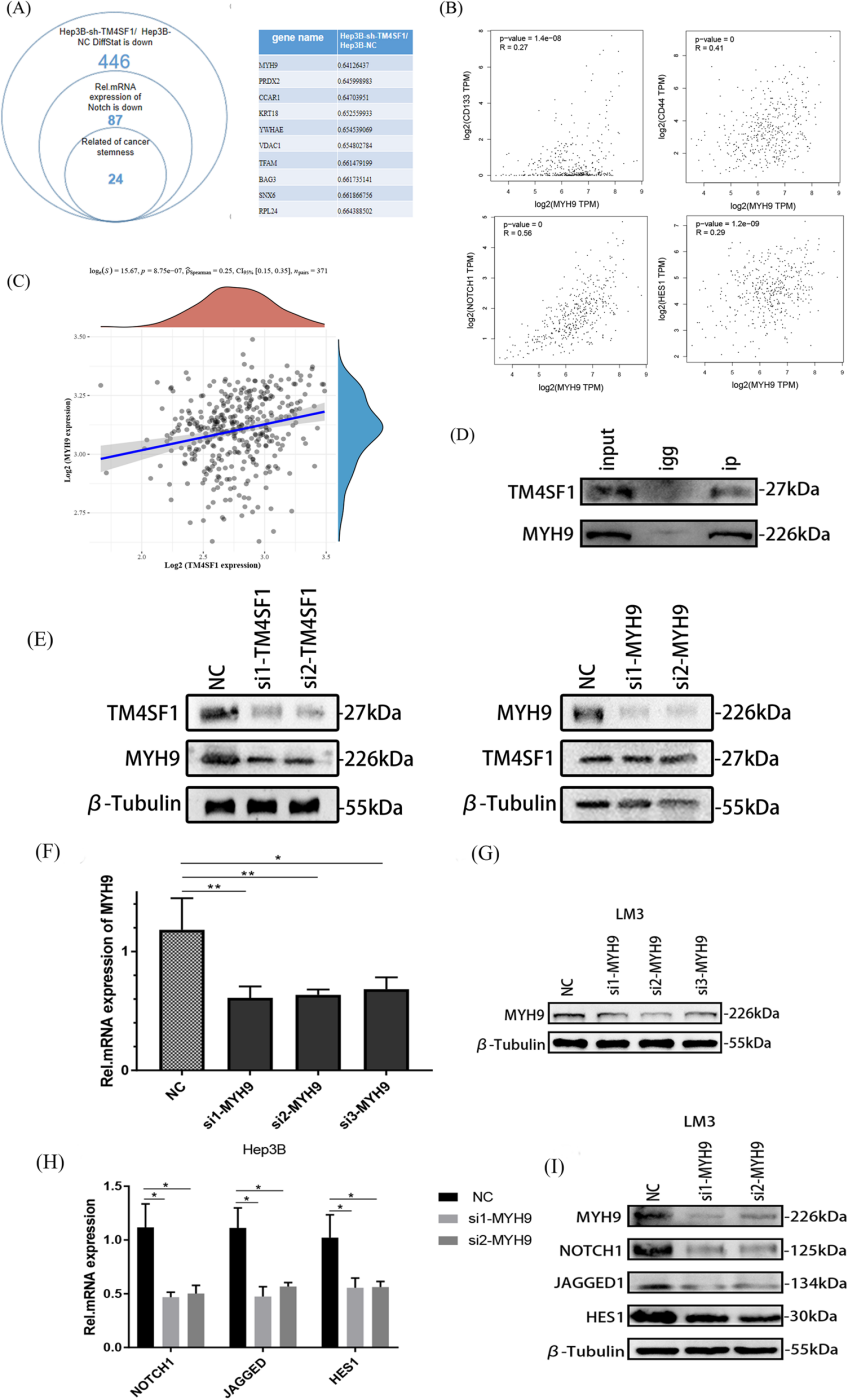
TM4SF1 could activate the NOTCH pathway through the upregulation of MYH9. **A** Genes whose expression changed after silencing TM4SF1 were screened via protein mass spectrometry, and the genes related to the NOTCH pathway and cancer stemness were gradually screened out via the correlation analysis of the GEPIA database. **B** Correlation of MYH9 with cancer stem–related molecules and the NOTCH pathway–related molecules via the correlation analysis of the GEPIA databases. **C** Using the TCGA database, the association between the expressions of TM4SF1 and MYH9 in 371 patients with HCC was examined. **D** Results of the Co-IP experiment showed that TM4SF1 could pull down MYH9. **E** WB analysis showed that the expression of MYH9 was down-regulated after TM4SF1 silence, but the expression of TM4SF1 was not affected after MYH9 silence. **F** Results of the qPCR analysis verified the expression efficiency after silencing MYH9. **G** Results of the WB analysis verified the expression efficiency after silencing MYH9. **H** The results of the qPCR test revealed that the expression level of molecules connected to the NOTCH pathway dropped when MYH9 was silenced. **I** The results of the WB analysis demonstrated that silencing MYH9 reduced the expression of molecules involved in the NOTCH pathway

**Fig. 6:**
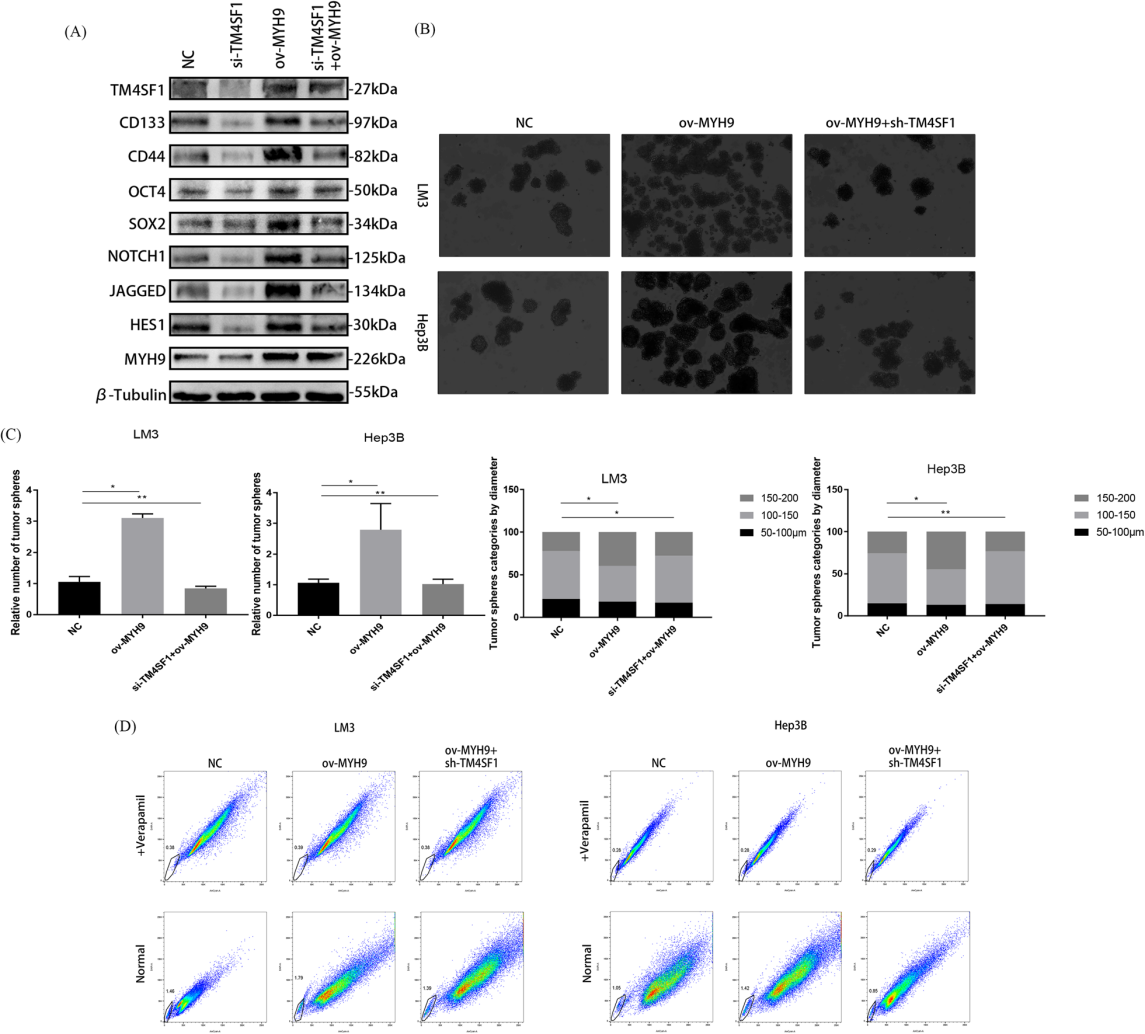
After the overexpression of MYH9, the effect of knocking down TM4SF1 on the cancer stemness of HCC cells could be recovered. **A** Results of the WB analysis showed that after the overexpression of MYH9, the decrease in the molecules related to the NOTCH pathway and cancer stemness caused by knocking down TM4SF1 could be restored in HCC cells. **B** Results of the experiment for the detection of side-population cells showed that after the overexpression of Myh9, the decrease in the proportion of stem cells caused by TM4SF1 knockout could be restored. **C** Experiment for the tumor sphere formation showed that after the overexpression of Myh9, the reduction in the number and size of tumor spheres caused by the silencing of TM4SF1 could be restored. Scale bars, 50 μm. **D** Comparison of the number of tumor spheres and the proportion of tumor spheres with different diameters

When considering the molecular mechanism by which TM4SF1 affected the cancer stemness in HCC cells, we first considered enriching the pathways of KEGG and GO for TM4SF1 using the GSEA technology. Considering 374 patients with liver cancer in the TCGA database as the samples, we enriched many TM4SF1-related pathways. Among the pathways related to cancer stemness, the NOTCH pathway attracted our attention because of its high correlation score (Fig. 4A). We detected the correlation between TM4SF1 and the common ligand JAGGED1, common receptor NOTCH1, and target gene HES1 of the NOTCH pathway via qPCR and WB, and found that the expression of JAGGED1, NOTCH1, and HES1 decreased after silencing TM4SF1 (Fig. 4B and C). RNA and protein extracted from primary tumor cells in nude mice were used for quantitative polymerase chain reaction (QPCR) and Western blotting (WB). The results showed that the expression of Notch pathway related molecules decreased in primary tumor cells of nude mice silenced with TM4SF1 (Fig. 4D and E). In order to further verify the regulatory effect of TM4SF1 on the NOTCH pathway, we purchased a targeted inhibitor RO4929097 of the NOTCH pathway. With the help of WB experiment, we found that after overexpression of TM4SF1, the inhibition effect of RO4929097 on the NOTCH pathway was reversed, and the NOTCH pathway was activated again (Fig. 4F). After confirming upregulation between TM4SF1 and the NOTCH pathway, we wondered how TM4SF1, as a single gene, activated the NOTCH pathway. Therefore, we detected using protein mass spectrometry the genes whose expression changed with the silencing of TM4SF1, and screened out some genes with significant correlation with the cancer stem–related molecules and NOTCH pathway molecules via the correlation analysis of the GEPIA database. When sequencing these differentially expressed genes from multiple species, we were surprised to find that the cytoskeleton gene MYH9 met our screening requirements and had a higher ranking (Fig. 5A and B). Hence, we hypothesized that TM4SF1 might affect the cancer stemness of HCC cells by regulating MYH9 and activating the NOTCH pathway. Through the analysis of TCGA database, we find that there is a high correlation between TM4SF1 and MYH9 (Fig. 5C). Meanwhile, through CO-IP and WB experiments, we found that TM4SF1, as a decoy protein, could fully and specifically bind to MYH9 in HCC cells. (Fig. 5D). In order to explore the specific regulatory relationship between TM4SF1 and MYH9 in liver cancer cells, we silenced TM4SF1 and MYH9 respectively, and observed the expression of another molecule by WB experiment. As a result, we found that when we silenced TM4SF1, the expression of MYH9 was significantly down-regulated, but when we silenced MYH9, the expression of TM4SF1 did not change significantly. Therefore, we believe that MYH9 can act as the downstream molecule of TM4SF1 in hepatocellular carcinoma cells and be subject to unilateral positive regulation from TM4SF1 (Fig. 5E). Then, we silenced MYH9 in LM3 cells, verify the silencing efficiency and detect the relationship between TM4SF1 and NOTCH pathway by qPCR and WB. The consequences indicated that the expression of JAGGED1, NOTCH1, and HES1 was downregulated after silencing MYH9 (Fig. 5F–I). Finally, we overexpressed MYH9 while knocking down TM4SF1. We confirmed via WB, detection of side-population cells, and tumor sphere formation that the overexpression of MYH9 could save the inhibition of cancer stemness in HCC cells caused by the silencing of TM4SF1 (Fig. 6A–D).

### TM4SF1 enhanced the lenvatinib resistance in HCC by promoting the cancer stemness in HCC cells

**Fig. 7:**
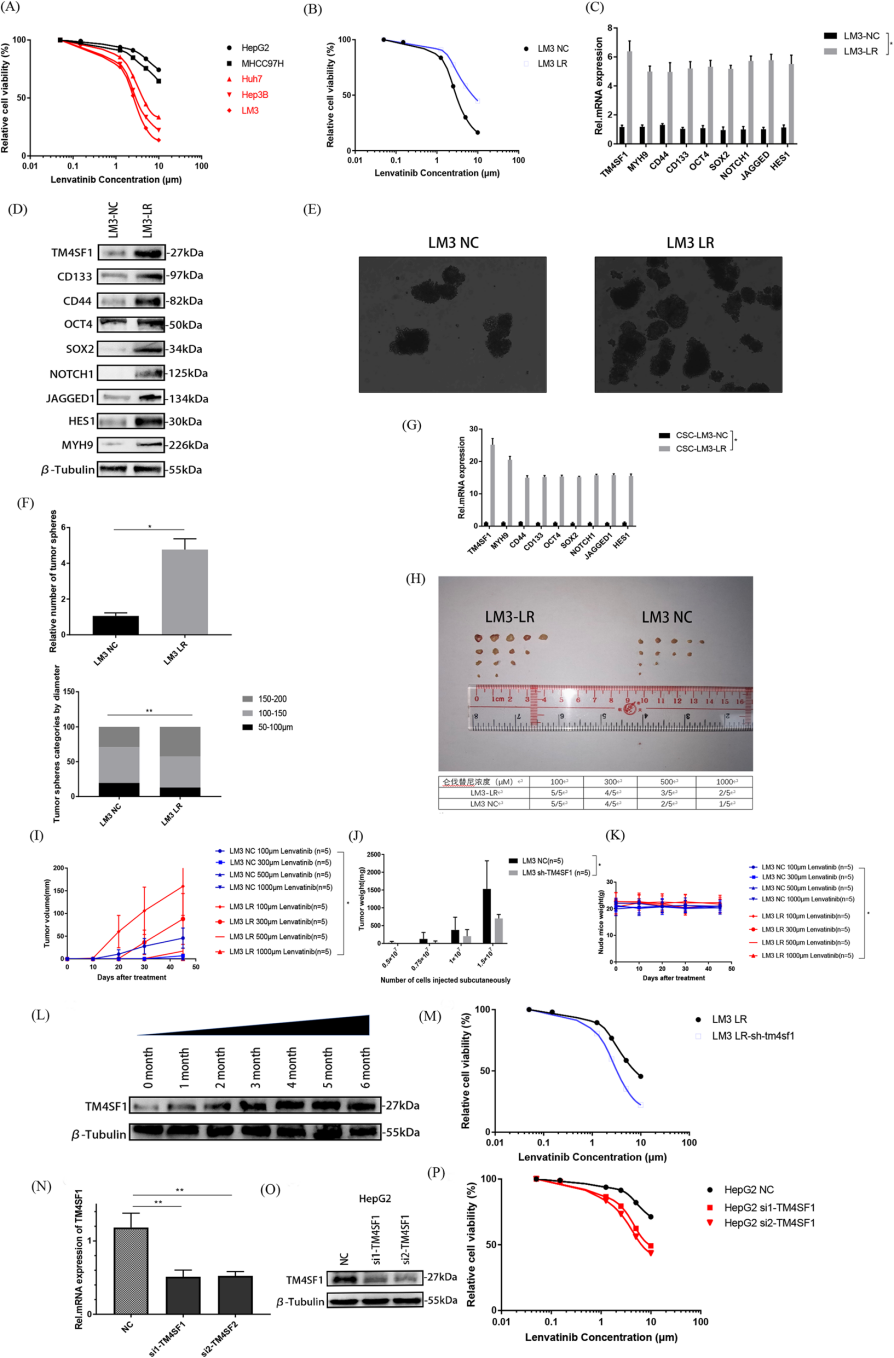
TM4SF1 could enhance the resistance of HCC to Lenvatinib by upregulating the activation of the NOTCH pathway using MYH9. **A** Determination of drug resistance in HCC cell lines to lenvatinib using CCK-8. **B** Using a concentration gradient approach, the Lenvatinib-resistant strain LM3-LR of LM3 cells was cultivated. WB analysis was used to identify the expression of TM4SF1 at various phases of the cultured LM3-LR cells. **C** Comparison of resistance to lenvatinib between LM3-LR and untreated groups using CCK-8. **D** The results of the qPCR were utilized to compare the expression of MYH9, molecules associated with cancer stem cells, and molecules connected to the NOTCH pathway in the LM3-LR and untreated groups. **E** Results of WB analysis were used to compare the expression of MYH9, cancer stem–related molecules, and the NOTCH pathway–related molecules in LM3-LR and untreated groups. **F** The tumor sphere production experiment revealed that the quantity and diameter of tumor spheres formed by LM3-LR cells were significantly higher than those of the group that had not received any treatment. Scale bars, 50 μm. **G** A comparison of the fraction of tumor spheres with various sizes and the quantity of tumor spheres. **H** Tumor spheres were used to extract RNA. After TM4SF1 was silenced, the qPCR analysis revealed that the expression of molecules linked to cancer stem cells, the NOTCH pathway, and MYH9 reduced. **I** Lenvatinib was administered intraperitoneally before the nude mice were split into the LM3-LR and untreated groups. The cultured nude mice's tumor was removed. **J** Statistical comparison of tumor volume between LM3-LR and untreated groups. **K** Statistical comparison of tumor weight between LM3-LR and untreated groups. **L** Statistical comparison of nude mice weight between LM3-LR and untreated groups. **M** CCK-8 findings revealed that after TM4SF1 was silenced, LM3-LR's resistance to Lenvatinib diminished. **N** Efficiency of silencing TM4SF1 in HepG2 was verified via the qPCR analysis. **O** Efficiency of silencing TM4SF1 in HepG2 was verified via the WB analysis. **P** CCK-8 findings demonstrated that TM4SF1 silencing reduced HepG2's resistance to Lenvatinib. ^*^P < 0.05; ^**^P < 0.01; ^***^P < 0.001

Currently, Lenvatinib is the treatment of choice for treating advanced-stage liver cancer; nevertheless, many patients have a poor therapeutic effect or a poor prognosis because of the resistance to Lenvatinib. We speculated that TM4SF1 affected not only the cancer stemness of HCC but also the Lenvatinib resistance in HCC because of the close relationship between cancer stemness and tumor drug resistance. We cultured hepatoma cell lines Hep3B, HepG2, LM3, Huh7, and MHCC97H in a cell medium with different concentrations of Lenvatinib, and used CCK-8 assay to detect the cell survival rate. The results showed that untreated HepG2 and MHCC97H cells had strong resistance to Lenvatinib, while untreated Hep3B, LM3, and Huh7 cells had poor resistance to Lenvatinib (Fig. 7A). We selected LM3, which was poorly resistant to Lenvatinib, and cultivated the drug-resistant strain LM3-LR having strong resistance to Lenvatinib with the increase in its concentration. We verified via the CCK-8 experiment that the resistance of LM3-LR cells to Lenvatinib was stronger than that of the untreated LM3 cells (Fig. 7C). The qPCR and WB detection showed that the expression of TM4SF1, cancer stem–related molecules, the NOTCH pathway–related molecules, and MYH9 in LM3-LR cells was higher than that in the control group (Fig. 7D and E). The tumor sphere formation experiment is also verified that the cancer stem function of LM3-LR cells was stronger than that of the control group (Fig. 7F and G). We extracted RNA from the tumor spheres, and through qPCR test, we found that the expression of TM4SF1 in the tumor spheres formed by LM3-LR was abnormally increased compared with the untreated group (Fig. 7H). In particular, the expression of TM4SF1 in LM3 cells increased with the increase in the Lenvatinib concentration (Fig. 7B). In order to further verify the accuracy of the previous experiment, we silenced TM4SF1 in LM3-LR cells. The CCK-8 experiment's findings demonstrated that the resistance of LM3-LR cells to lenvatinib decreased (Fig. 7M). Similarly, we also silenced TM4SF1 in HepG2 cells with strong Lenvatinib resistance (Fig. 7N and O), and found that the resistance of HepG2 with silenced TM4SF1 to Lenvatinib also decreased compared with that of the untreated group (Fig. 7P).

We also performed the in vivo drug resistance test on subcutaneous tumor formation in nude mice based on the aforementioned findings (Fig. 7I), and found that the tumor size and weight of nude mice decreased gradually with the increase in the lenvatinib concentration injected through the abdominal cavity. However, when injected with the same Lenvatinib concentration, the volume and weight of subcutaneous tumors of nude mice injected with LM3-LR cells were larger than those of the control group injected with untreated LM3 cells (Fig. 6J and K). Finally, in order to prove the safety of Lenvatinib in nude mice and the stability of treatment, we weighed the nude mice regularly. The findings demonstrated that during Lenvatinib treatment, there was no discernible variation in body weight between the two groups (Fig. 7L).

## Discussion

Although various treatments are available for liver cancer, the main treatment to date is hepatectomy or liver transplantation via clinical surgery [26, 27]. Only when liver cancer is in its early stages may surgery be utilized to treat it effectively. Due to the absence of distinctive signs in the early stages of liver cancer, many individuals have advanced stages of the disease by the time they are diagnosed [28]. Hence, treating patients with advanced-stage liver cancer are particularly difficult and quite challenging. Therefore, the occurrence and development of liver cancer and its molecular mechanism need to be urgently investigated to find the clinical treatment target of liver cancer. Numerous studies have shown that TM4SF1 is strongly associated with the occurrence and progression of cancer [20–22]. Zeng and colleagues showed in 2021 that TM4SF1 played a role in advancing the proliferation, invasion, and metastasis of HCC [23]. CSCs are crucial in cancer, according to a vast number of studies. Inducing the creation and growth of primary tumors is a crucial biological activity, but it also plays other significant roles [5, 6]. but also a driving factor to promote cancer drug resistance [10–12]. However, few reports exist on the relationship between TM4SF1 and cancer stemness. A recent study showed that TM4SF1 promoted proliferation, invasion, metastasis, and stemness in colon cancer [24]. These studies suggested that TM4SF1 played a promoting role in various cancers and might have a potential link with cancer stemness. However, the effect of TM4SF1 on the stemness of HCC and its related mechanism have not been reported. Since TM4SF1 is strongly expressed in liver cancer cells and tissues compared to normal liver cells and tissues, we investigated its impact on the stemness of HCC in this work. We also confirmed that the expression of TM4SF1 negatively correlates with patient prognosis. The experiment of subcutaneous tumorigenesis in nude mice and the detection of RNA and protein levels in primary cells prepared from tumors in nude mice also confirmed this finding. We also discovered that the expression of stemness-related molecules CD44, CD133, OCT4, and SOX2 also decreased with the decrease in the expression of TM4SF1. The tumor sphere formation test and side-population cell detection also showed that the expression level of TM4SF1 was positively correlated with the cancer stemness of HCC. These results suggested that TM4SF1 might be a potential factor affecting the stemness of HCC.

The NOTCH signaling pathway was first found in *Drosophila melanogaster* and plays an major role in embryonic development [29]. In this pathway, the extracellular ligand JAGGED1 has the main role: when activated, it binds to the receptor NOTCH1–4 on the cell membrane to form the intracellular signal transduction molecule NICD and finally acts on the nuclear target gene HES1 [30]. The NOTCH pathway has been associated to the occurrence and progression of cancer in recent years [31, 32]. In 2013, Jia Lu et al. found that the activation of the NOTCH pathway promoted cancer stemness in patients with rectal cancer [33]. However, in a 2021 study, Guenter and his collaborators found that the NOTCH pathway positively mediates cancer stemness in patients with thyroid cancer [34]. The NOTCH pathway is crucial for the cancer's stemness. In this research, we took TM4SF1 as the research object and enriched a series of related pathways using the GSEA technology. The NOTCH pathway aroused our interest because of its close and high correlation with cancer stemness. We verified via qPCR and WB experiments that the expression of TM4SF1 was positively correlated with the expression of the NOTCH pathway ligand JAGGED1, receptor NOTCH1, and target gene HES1 in HCC cells, meanwhile, with the help of the NOTCH pathway targeted inhibitor RO4929097, we found that overexpression of TM4SF1 could reverse the inhibitory effect of RO4929097 on the NOTCH pathway. We hypothesized that TM4SF1 might promote cancer stemness in HCC via activating the NOTCH signaling pathway.

The heavy chain of non-muscle myosin IIA, or MYH9, is a protein of 1960 amino acids, which is an important constituent of the non-muscle cytoskeleton [35]. In recent years, many researchers have provided strong evidence that MYH9 plays a key role in the initiation and progression of a number of malignancies [36–39]. Lin X and his collaborators showed in a 2020 study that silencing MYH9 could inhibit cancer stemness in HCC [40]. These studies demonstrated that the cancer stemness in patients with liver cancer may be significantly impacted by MYH9. We screened out some genes, using protein mass spectrometry, whose expression changed with the silencing of TM4SF1 in HCC cells. Also, we tested the correlation between these genes and cancer stem–related molecules and the NOTCH pathway–related molecules using the GEPIA database. After screening and arranging the data, we found that MYH9 had good performance in differentially expressed genes from multiple species and their correlation rankings. We verified via Co-IP and WB analysis that TM4SF1 could pull down MYH9, Meanwhile, WB experiment also verified that as a downstream molecule, M MYH9 is positively regulated by TM4SF1 and MYH9 expression in HCC cells was favorably connected with the NOTCH pathway's associated components. Therefore, we hypothesized that TM4SF1 activated the NOTCH pathway by positively regulating MYH9, which in turn promoted cancer stemness in HCC.

Lenvatinib is an oral small-molecule inhibitor of multi-receptor tyrosine kinase that has received approval for use in many nations as the first-line therapy for advanced-stage or irreversible HCC [13]. Lenvatinib, as a traditional first-line treatment drug, has made an outstanding contribution to the treatment of patients with advanced-stage liver cancer [41]. However, the tolerance and poor prognosis of some patients are also the disadvantages of Lenvatinib [25, 42, 43]. In previous studies, some scholars made many speculations about the principle of lenvatinib resistance, but still many mysteries remain unsolved. After verifying the promoting effect of TM4SF1 on the cancer stemness of HCC, we speculated based on the close relationship between cancer stemness and tumor drug resistance, that TM4SF1 could enhance the Lenvatinib resistance in HCC cells by promoting the cancer stemness in HCC. The strain LM3 with poor resistance to Lenvatinib was screened using the CCK-8 test, and the resistant strain LM3-LR with strong tolerance to Lenvatinib was cultivated. A series of tests were carried out to verify the effect of drug resistance. Subsequently, we found that the expression of TM4SF1, MYH9, and the NOTCH pathway–related molecules increased in LM3-LR cells. We silenced TM4SF1 in HepG2 and LM3-LR cells, which were more tolerant to Lenvatinib, and found that their resistance to Lenvatinib decreased again. We carried out a subcutaneous tumor formation experiment in nude mice to confirm the accuracy of this result. The results also supported our previous hypothesis.

We verified that TM4SF1 could activate the NOTCH signaling pathway by positively regulating MYH9, which ultimately promoted the cancer stemness in HCC and enhanced the drug resistance of HCC cells to Lenvatinib (Additional file 3: Fig. S2). As a result, TM4SF1 may represent a promising target for the clinical treatment of HCC and contribute significantly to the enhancement of Lenvatinib's therapeutic effects in patients with advanced-stage HCC. First, in order to offer a theoretical foundation for a more precise clinical targeted therapy for HCC, we should conduct a more in-depth investigation into the molecular mechanism by which TM4SF1 activates the NOTCH pathway. In order to improve the curative effect of Lenvatinib and develop new medications to improve the prognosis in patients with HCC, additional research should be done to explore the precise mechanism of TM4SF1 affecting lenvatinib resistance in HCC. This will increase the effectiveness of medications in clinical trials while maintaining safety.

## Supplementary Information

**Additional file 1: Table S1.** The sequences used in this study. **Table S2.** The primers used in this study.

**Additional file 2: Fig. S1.** The bioinformatics databases showed that TM4SF1 is highly expressed in HCC and correlated with poor prognosis. **A** Research on the TIMER2.0 database showed that hepatocellular cancer had significant levels of TM4SF1 expression. **B** Information from the GEPIA database revealed that the expression of TM4SF1 was higher in tissues with hepatocellular carcinoma than in healthy tissues. **C** Analysis of the GEPIA database and TIMER2.0 database showed that the cumulative survival rate, overall survival rate and disease-free survival rate of patients with low TM4SF1 expression were better than those with high TM4SF1 expression. **D** Expression of TM4SF1 in hepatocellular carcinoma cell lines was analyzed using the TCGA database.

**Additional file 3: Fig. S2.** Schematic diagram of the molecular mechanism of TM4SF1 upregulates MYH9 to activate the NOTCH pathway to promote cancer stemness and Lenvatinib resistance in HCC.

## Data Availability

1. The “expression of TM4SF1 in HCC” and “the cumulative survival rate” data that support the findings of this study are available in “TIMER2.0”, “http://timer.cistrome.org/”. 2. The “expression of TM4SF1 in HCC” “intermolecular correlation analysis” and “the overall survival rate and disease-free survival rate” data that support the findings of this study are available in “GEPIA”, “http://gepia2.cancer-pku.cn/#index”. 3. The “expression of TM4SF1 in HCC cell lines” and “stemness index of TM4SF1” data that support the findings of this study are available from “TCGA”, “https://www.cancer.gov/about-nci/organization/ccg/research/structural-genomics/tcga”. 4. The “GSEA Pathway enrichment” data that support the findings of this study are available from “KEGG”and “GO”, “https://www.kegg.jp/” and “http://geneontology.org/”.
