# Supplementary material for: TM4SF1 upregulates MYH9 to activate the NOTCH pathway to promote cancer stemness and lenvatinib resistance in HCC
Source: Biol Direct. 2023 Apr 17;18:18. doi: 10.1186/s13062-023-00376-8 (PMC10111829; doi:10.1186/s13062-023-00376-8)
Supplement: Supplementary file 1 — Additional file 1: Table S1. The sequences used in this study. Table S2. The primers used in this study. [file 13062_2023_376_MOESM1_ESM.docx]

**Supplementary Table1. The sequences used in this study.**

| Gene | Target sequences |
| --- | --- |
| TM4SF1 | GUGUCUUAUUCAAGUAAUAAA |
|  | CAUCUUGUGUCUUAUUCAAGU |
|  | GGCUCUUGGUGGAAUUGAAUU |
| MYH9 | GCAAGACCGAGAAGAUCAAUC |
|  | GGACCGAGAAGAAGCUGAAGG |
|  | CUGUCAAGUCCAAGUACAAGG |

**Supplementary Table2. The primers used in this study.**

| Primers name |  | sequences |
| --- | --- | --- |
| TM4SF1 | Forward | GTGGAGGAAATTGTGGCTGT |
|  | Reverse | CGTTCATGGTGATCCAACTG |
| CD133 | Forward | AGTCGGAAACTGGCAGATAGC |
|  | Reverse | GGTAGTGTTGTACTGGGCCAAT |
| CD44 | Forward | TCTACAAGCACAATCCAGGCA |
|  | Reverse | TGGTATGAGCTGAGGCTGC |
| OCT4 | Forward | AGTTCTTGGGAATGGACGGC |
|  | Reverse | TCCGAATACTCCTGCACCGAT |
| SOX2 | Forward | TGGACAGTTACGCGCACAT |
|  | Reverse | CGAGTAGGACATGCTGTAGGT |
| NOTCH1 | Forward | GAGGCCTGCGTCTGTGG |
|  | Reverse | CATTGTCCAGGGGTGTCAGG |
| JAGGED1 | Forward | GGCCGAGGTCCTATACGTTG |
|  | Reverse | ACACAAGGTTTGGCCTCACA |
| HES1 | Forward | ATGCTTAGTGGAGCTACGCC |
|  | Reverse | TGCTCCACTGTCAAGTGCAA |
| MYH9 | Forward | CCAGGCCAAAGAGAACGAGA |
|  | Reverse | CTAACGCCAGGGCTCCTTTG |
| β-Tubulin | Forward | CTCTTTCCGTCCACCAGCTT |
|  | Reverse | CAAATACACGAGAGCAGGGC |
