# Supplementary figures and images for: TM4SF1 upregulates MYH9 to activate the NOTCH pathway to promote cancer stemness and lenvatinib resistance in HCC
Source: Biol Direct. 2023 Apr 17;18:18. doi: 10.1186/s13062-023-00376-8 (PMC10111829; doi:10.1186/s13062-023-00376-8)

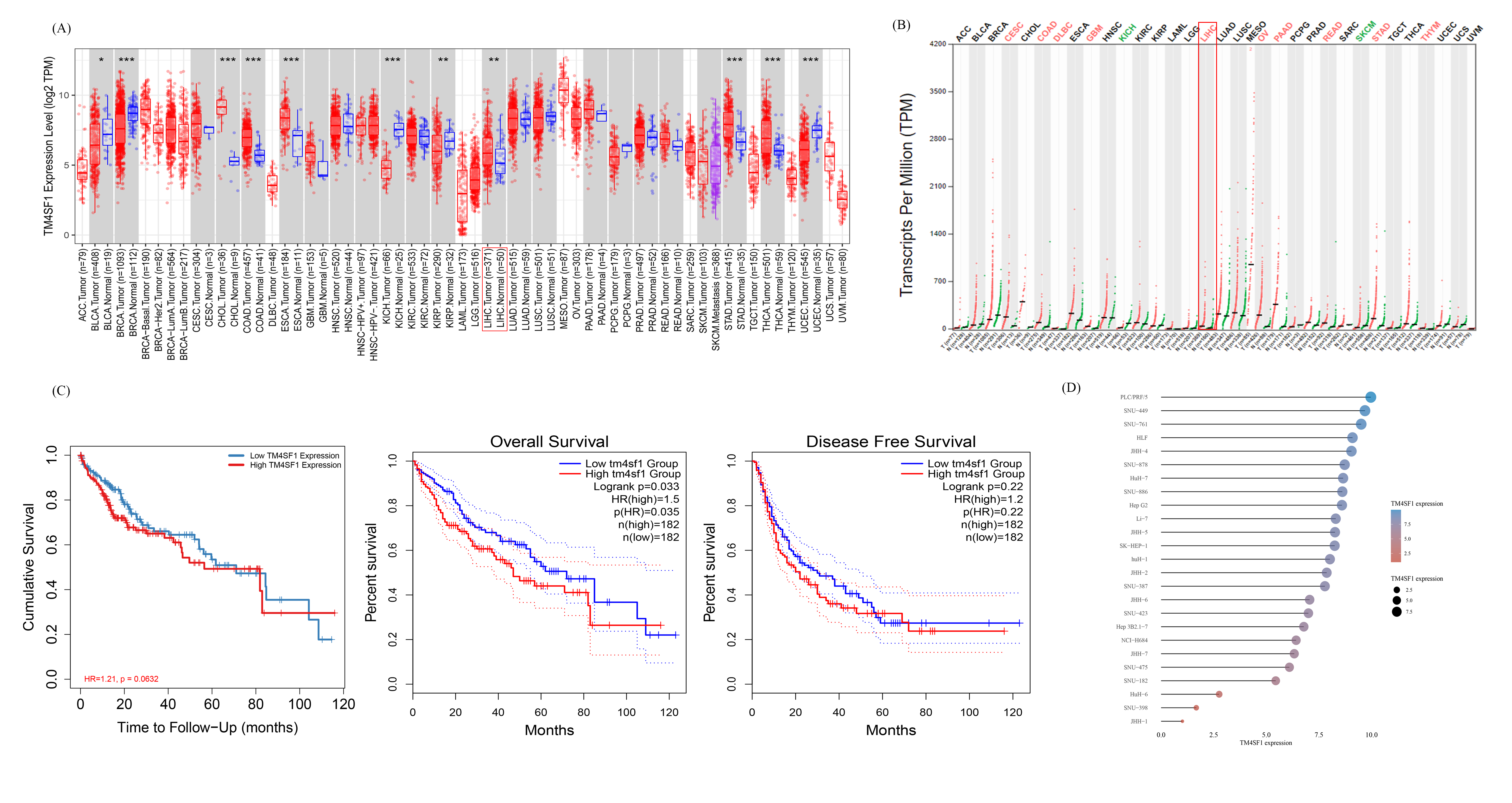

Supplement: Supplementary file 2 — Additional file 2: Fig. S1. The bioinformatics databases showed that TM4SF1 is highly expressed in HCC and correlated with poor prognosis. A Research on the TIMER2.0 database showed that hepatocellular cancer had significant levels of TM4SF1 expression. B Information from the GEPIA database revealed that the expression of TM4SF1 was higher in tissues with hepatocellular carcinoma than in healthy tissues. C Analysis of the GEPIA database and TIMER2.0 database showed that the cumulative survival rate, overall survival rate and disease-free survival rate of patients with low TM4SF1 expression were better than those with high TM4SF1 expression. D Expression of TM4SF1 in hepatocellular carcinoma cell lines was analyzed using the TCGA database. [file 13062_2023_376_MOESM2_ESM.tif]
