# Supplementary material for: TM4SF1 upregulates MYH9 to activate the NOTCH pathway to promote cancer stemness and lenvatinib resistance in HCC
Source: Biol Direct. 2023 Apr 17;18:18. doi: 10.1186/s13062-023-00376-8 (PMC10111829; doi:10.1186/s13062-023-00376-8)

**Supplementary Figure 2. Schematic diagram of the molecular mechanism of TM4SF1 upregulates MYH9 to activate the NOTCH pathway to promote cancer stemness and Lenvatinib resistance in HCC**


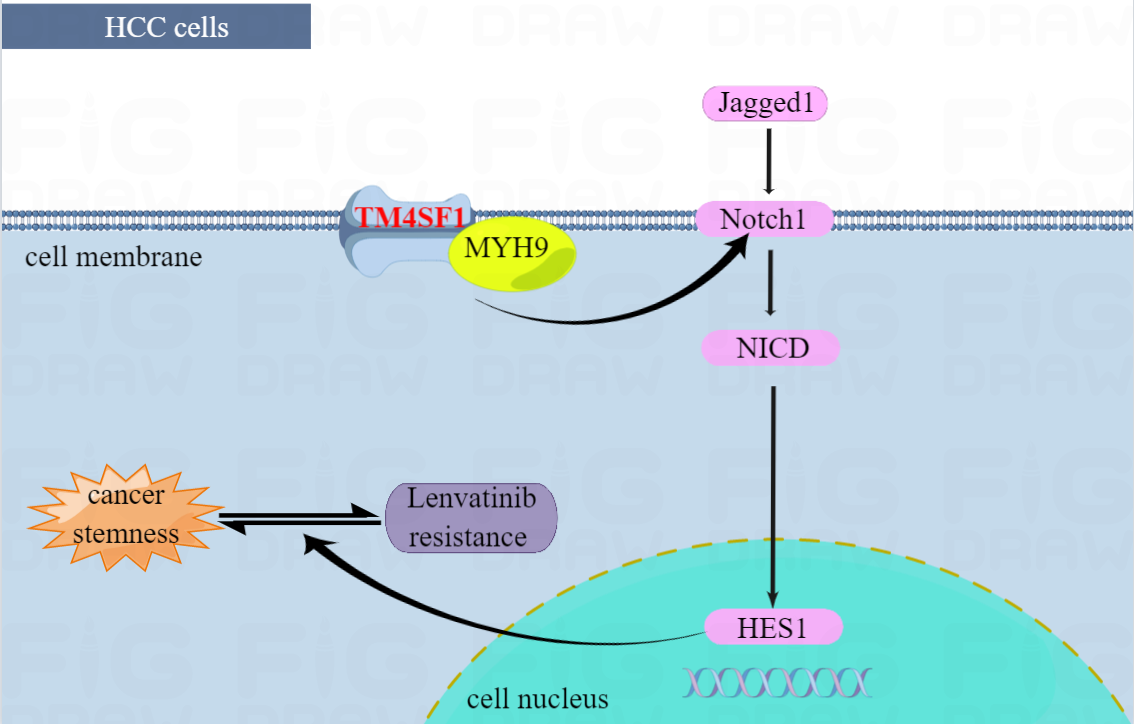

Supplement: Supplementary file 3 — Additional file 3: Fig. S2. Schematic diagram of the molecular mechanism of TM4SF1 upregulates MYH9 to activate the NOTCH pathway to promote cancer stemness and Lenvatinib resistance in HCC. [file 13062_2023_376_MOESM3_ESM.docx]
